# Intervention for job burnout reduction among a sample of Nigerian lecturers

**DOI:** 10.1097/MD.0000000000033425

**Published:** 2023-04-14

**Authors:** Chiedu Eseadi, Zadrian Ardi, Vera Victor-Aigbodion, Roland Ndille, Hero Usiomoefo Obasuyi, Shulamite Ebere Ogbuabor

**Affiliations:** a Department of Educational Psychology, University of Johannesburg, Johannesburg, South Africa; b Department of Guidance and Counseling, Universitas Negeri Padang, Padang, Indonesia; c Department of History, University of Buea, Buea, Cameroon; d Department of History and International Studies, University of Nigeria, Nsukka, Nigeria; e Department of Educational Foundations, University of Nigeria, Nsukka, Nigeria.

**Keywords:** history lecturers, job burnout, lecturers’ burnout, online psychological intervention, online REBT, REBT

## Abstract

One of the major issues facing Nigerian university lecturers is job burnout. In the current study, Nigerian university history lecturers were examined in respect of job burnout prior to and after an online psychological intervention that followed the rational emotive behavior therapy (REBT) principles and practice.

**Methods:**

In this study, a group randomized controlled trial approach was used, and only 80 university history lecturers with high burnout levels were included. We had 40 history lecturers participating in an online intervention group and 40 history lecturers participating in the control group. A questionnaire – Oldenburg Burnout Inventory – was used to collect data about job burnout.

**Results:**

After the history lecturers underwent the online psychological intervention (online REBT), significant reductions in mean job burnout were recorded (*F*(1, 78) = 5756.11; *P* < .001). Findings show a statistically significant effects of time on burnout scores of history lecturers [*F*(2156) = 1323.69, *P* < .001, ω^2^ = 0.92]. There was also a significant group and time interaction effect on the participants’ burnout scores [*F*(2156) = 1323.69, *P* < .001, ω^2^ = 0.91].

**Conclusion:**

University history lecturers can benefit from online psychological intervention that targets job burnout reduction. The current study paves way for future studies to validate the efficacy of online REBT intervention among other employees who struggle with burnout problem.

## 1. Introduction

The increasing workplace demands in the university environments can greatly impact history lecturers’ career and mental toughness. Besides teaching, grading, and supervising students, the lecturers are expected to carry out research, engage in article writing, and team up with their colleagues^[1]^ Due to prolonged workplace demands and stress, history lecturers can experience extreme exhaustion, reduced productivity, and depersonalization, referred to as burnout. Burnout is common among university lecturers in Nigeria.^[1–3]^ Burnout among history lecturers can have a negative effect on both the lecturers’ and students’ outcomes. Burnout can negatively affect a person’s physical and mental well-being, impair their ability to produce quality work, and limit their ability to grow professionally.^[4]^ Several studies, including Burke et al,^[5]^ Hakanen et al,^[6]^ and Vesty et al,^[7]^ have shown that job burnout is associated with deteriorating emotional, physical, and mental health, a lack of work engagement and commitment, and a high level of despair and fatigue. The results of current studies continue to show the negative effects of job burnout among employees in terms of low satisfaction, high turnover intentions, and poor quality of teacher-student relationships, especially during the Covid-19 pandemic.^[8–11]^

Due to the Covid-19 pandemic, the number of personal contacts dwindled and distance became more prevalent, leading to the necessity of online intervention to address job burnout issues among history lecturers. In this study, telegram was used to deliver the intervention. Instant messaging, large communication files, broadcast channels, and groups are all supported in this application and it has a swift connection speed, a large user capacity, and can be accessed via several internet connected devices.^[12]^ The Internet has enabled online interventions to take place and evolve in many ways over the years.^[13]^ Online interventions have been successfully used by researchers in the treatment of depression, anxiety, or problematic substance use.^[14–17]^

Theoretically, burnout symptoms onset is thought to be a result of unrealistic beliefs in rational emotive behavior therapy (REBT)’s perspective.^[18,19]^ According to the theory of REBT, burnout behavior is related to irrational beliefs and maladaptive thinking patterns.^[20,21]^ The use of REBT can improve mental toughness and resilience in individuals by targeting both their maladaptive cognitions and disturbed emotions.^[22–24]^ A number of studies have demonstrated the effectiveness of REBT interventions in reducing job burnout.^[25–28]^ In a recent study by Nwabuko et al,^[29]^ the use of a REBT program reduced burnout symptoms among school teachers. Upon conducting a literature review, we found that research on the effectiveness of REBT treatment for burnout among history lecturers in Nigerian universities is lacking. Using online REBT intervention, this study in terms of its objective, is examining whether it can reduce job burnout among history lecturers in Nigeria. The online REBT intervention was hypothesized to reduce job burnout among history lecturers.

## 2. Methods

The Education Faculty body responsible for research ethics at the University of Nigeria approved this research. Before the intervention began, each history lecturer signed a consent form electronically. Eighty history lecturers from federal and state universities in the Southeastern geographical region of Nigeria with high levels of burnout as determined by the Oldenburg Burnout Inventory were deemed eligible for this study. This study set criteria for eligibility, including: the participant must work in either a federal or state university, must show signs of burnout as measured by an online version of the Oldenburg Burnout Inventory, participants must not have mental illnesses, must have a functioning Smartphone with a Telegram app and a working email. The research included only participants who met these eligibility criteria.

The Oldenburg Burnout Inventory consists of 16 questions originally written in German but translated into English by Demerouti et al.^[30]^ This was used to determine how disengaged and exhausted the lecturers were. In terms of disengagement and exhaustion, each contained 8 questions, 4 positive questions and 4 negative questions, with the negative questions reversible in order to measure lecturers’ degree of burnout. The 4-point scale ranged between 1 (strongly agree), 2 (agree), 3 (disagree), and 4 (strongly disagree). Lecturers with high scores on the inventory were involved in this study. Oldenburg Burnout Inventory was reported to be reliable when measuring exhaustion (Cronbach’s alpha of 0.87) and disengagement (Cronbach’s alpha of 0.83) by Igbokwe et al.^[31]^ According to our analysis, the Cronbach’s alpha score for disengagement is 0.85, and the Cronbach’s alpha score for exhaustion is 0.84.

**Figure 1.**
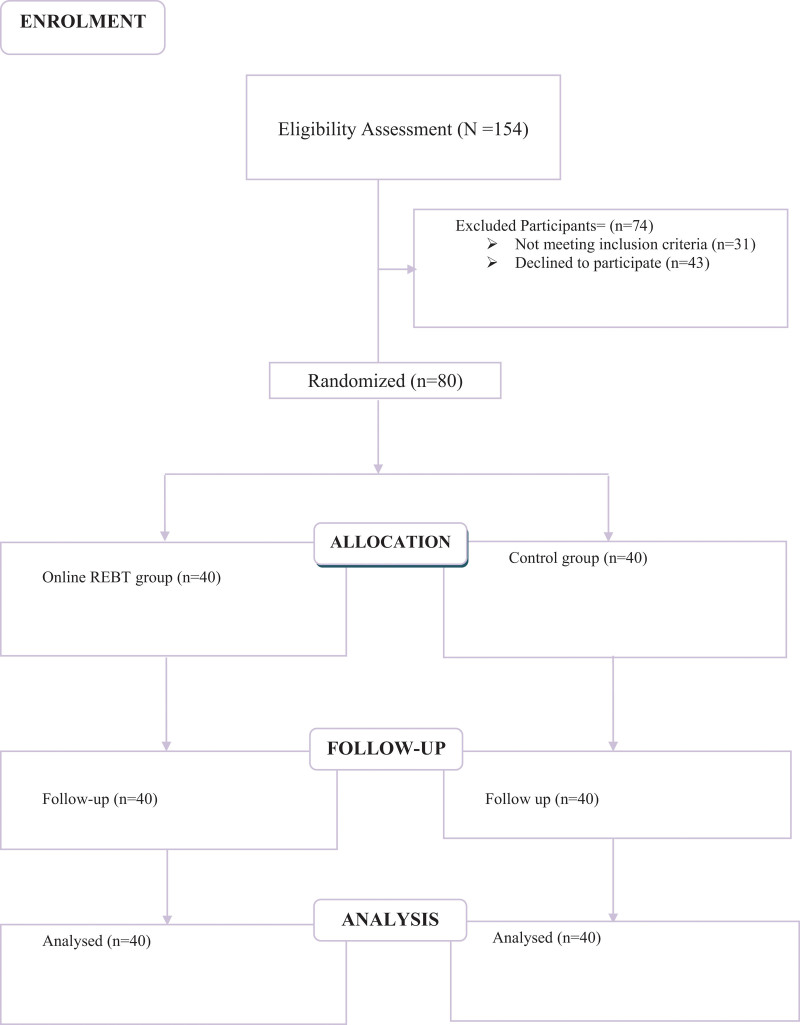
Participant flowchart.

This study used a group randomized controlled trial approach consisting of simple randomization. During July to December 2021, the online intervention and nonintervention groups were assessed at pretest, posttest, and follow-up. The study assessed 154 history lecturers and their eligibility was determined using the Oldenburg Burnout Inventory. At the end of baseline assessment, we had 40 history lecturers participating in the online intervention group and 40 history lecturers participating in the control group (see Fig. 1). This was accomplished using the random allocation software developed by Saghaei.^[32]^ Telegram groups were created for participants in the experimental and control arms. In the online intervention group, participants were assigned a 75-minute weekly REBT burnout treatment program for 10 weeks; while in the control group, participants were only offered daily inspirational messages through Telegram. The online intervention used in this study was based on a digitalized version of the Rational Emotive Behaviour Therapy Burnout treatment manual.^[18]^

First to fourth weeks of the program involved job burnout assessment, relationship building between therapists and participants, education of participants regarding REBT, treatment norm and basic rules of therapy, and creation of a problem list. From week 5 to 8, REBT techniques were used to challenge and weaken the participants’ burnout beliefs, and to develop and increase participants’ rational beliefs. During weeks 9 and 10, the participants were prepared to assume the role of self-therapists in managing burnout; problem-solving skills, cognitive hardiness and resilience against job burnout were also improved. The research therapists provided chats and audio messages in each online session. After 3 months, there was a 3-week follow-up. The study data were analyzed via repeated measures ANOVA in the JASP statistical program.

## 3. Results

Data in Table 1 show the mean and standard deviation of the treatment and control groups across Time 1 (pretest), Time 2 (post-test), and Time 3 (follow-up) evaluations. At Time 1, the treatment group had a mean score of 58.33 (SD = 1.54), while the control group had a mean score of 58.60 (1.71). This indicates that both the treatment and control groups had high level of burnout before the online intervention. At Time 2, the burnout score of the treatment group reduced to 33.35 (SD = 2.42), compared to the control group that had mean score of 57.45 (SD = 1.68). Further, at Time 3, the treatment group had reduced burnout score of 32.08 (SD = 2.06) compared to the control group that still showed high level of burnout with mean of 58.17 (SD = 1.34). These outcomes, suggest that online intervention reduced burnout symptoms among history lecturers. This finding is strengthened by repeated measure ANOVA analyses presented in Tables 2.

**Table 1:** Descriptive statistics for the history lecturers’ burnout scores.

Time	Group	Mean	SD	N
Time 1	Control	58.60	1.71	40
	Treatment	58.33	1.54	40
Time 2	Control	57.45	1.68	40
	Treatment	33.35	2.41	40
Time 3	Control	58.17	1.34	40
	Treatment	32.08	2.06	40

N = number of participants, SD = standard deviation.

**Table 2:** Simple main effects – group.

Time	Sum of squares	df		Mean square	*F*	*P*
Time 1	1.51		1	1.51	0.57	.45
Time 2	11616.20		1	11616.20	2688.62	<.001
Time 3	13624.20		1	13624.20	4530.75	<.001

Data in Table 2 presents the main effect of group. Data presented in Table 2 indicate that the intervention and control groups had a non significant difference in their burnout scores at Time 1 (*P* = .45). This shows that before the intervention, both the experimental and control groups had equally high level of burnout. The main effect of the intervention on the treatment groups lead to a significant difference in the burnout scores of the treatment and control groups at Time 2 (*P* < .001), and Time 3 (*P* < .001).

Table 3 showed the between subject effect of the treatment intervention on the burnout rating of the participants. The table indicates a significant between subject effect of the online intervention on the treatment and control groups (*F*(1, 78) = 5756.11; *P* < .001), with a high effect size as indicated by the high value of omega squared (ω^2^ = 0.97). This means that online intervention significantly reduced burnout among the participants in the treatment group over those in the control group.

**Table 3:** Repeated measure of the between subjects effects of the treatment on the groups.

Cases	Sum of squares	df	Mean square	*F*	*P*	ω^2^
Group	16984.84	1	16984.84	5756.11	<.001	0.97
Residuals	230.16	78	2.95			

**Figure 2.**
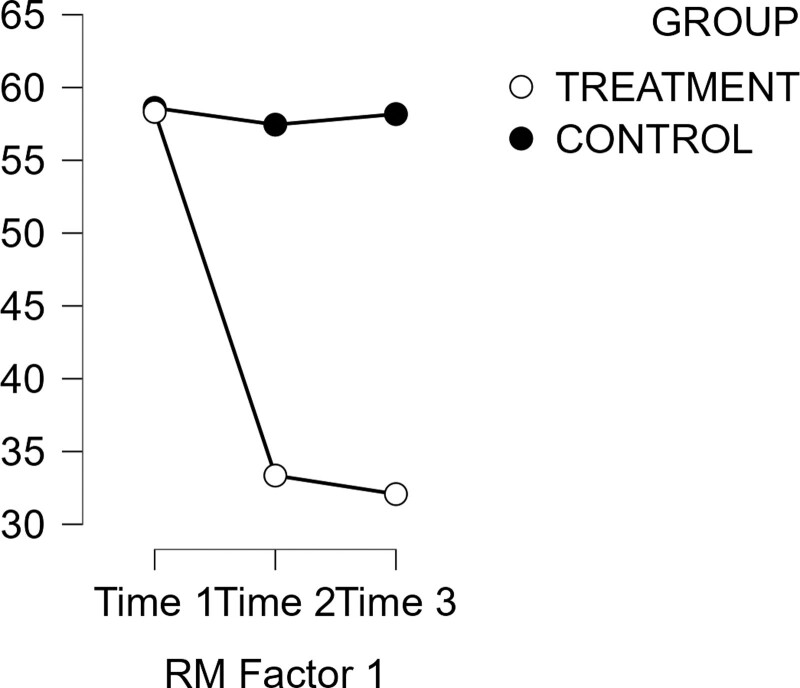
Interaction effect of time and group on burnout of history lecturers.

Data in Table 4 further show a statistically significant effects of time on burnout scores of history lecturers [*F*(2156) = 1323.69, *P* < .001, ω^2^ = 0.92]. There is also a significant group and time interaction effect on the participants’ burnout scores [*F*(2156) = 1323.69, *P* < .001, ω^2^ = 0.91]. Figure 1 further shows the interaction effect of group and time on burnout scores of history lecturers. As shown in the figure, burnout scores of the treatment group decreased significantly from Time 1 to Time 2, but did not change significantly from Time 2 to Time 3. On the other hand, the burnout scores of the control group did not vary significantly across Times 1-2, and Times 2-3 (see Fig. 2; Table 5).

**Table 4:** Repeated measure of the within subjects effects of the treatments on group.

Cases	Sum of squares	df	Mean square	*F*	*P*	ω^2^
Time	9295.82	2	4647.91	1323.69	<.001	0.92
Time ✻ GROUP	8257.07	2	4128.54	1175.78	<.001	0.91
Residuals	547.77	156	3.51			

**Table 5:** Post hoc comparisons – time.

			95% CI for mean difference					95% CI for Cohen’s d		
		Mean difference	Lower	Upper	SE	*t*	Cohen’s d	Lower	Upper	*P*_bonf_
Time 1	Time 2	13.06	9.71	16.41	1.37	9.53	7.16	5.73	8.60	<.001*
	Time 3	13.34	9.71	16.97	1.48	8.98	7.31	5.85	8.78	<.001*
Time 2	Time 3	0.28	−0.49	1.04	0.31	0.88	0.15	−0.24	0.54	1.00

Computation of Cohen’s d based on pooled error.

*P* value and confidence intervals adjusted for comparing a family of 3 estimates (confidence intervals corrected using the bonferroni method).

Results are averaged over the levels of: Group.

* *P* < .001.

**Figure 3.**
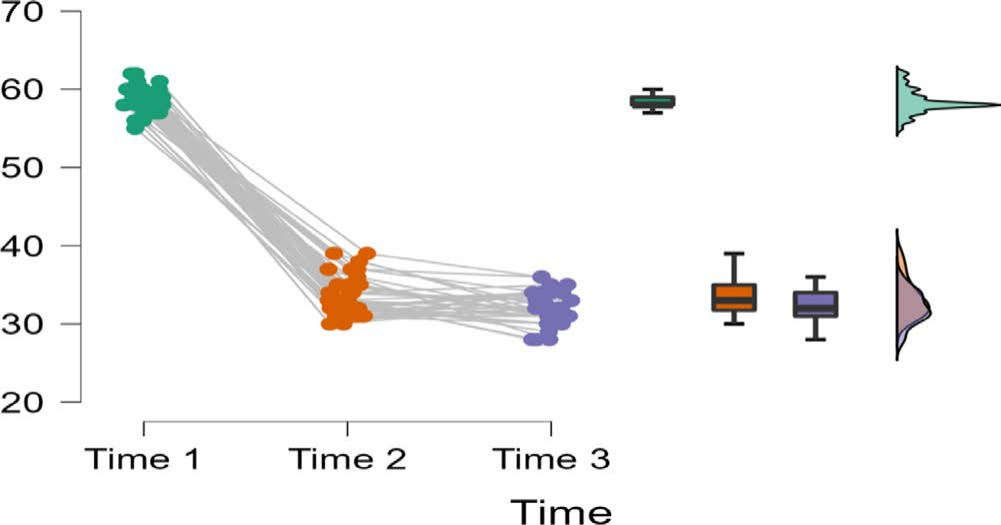
Raincloud plots burnout scores across time for treatment group.

A Post Hoc comparison of the effect of Time on the burnout scores of the intervention group is shown in Table 5. The Table indicates that the difference in burnout scores of the group were significant between Times 1-2 (Mean difference = 13.06; *t* = 9.53; *P*_bonf_ < .001), and Times 1-3 significant (Mean difference = 13.34; *t* = 8.98; *P*_bonf_ < .001), but was not significant between Times 2-3 (Mean difference = 0.28; *t* = 0.88; *P*_bonf_ > .05). This outcome is illustrated in Figure 3.

**Figure 4.**
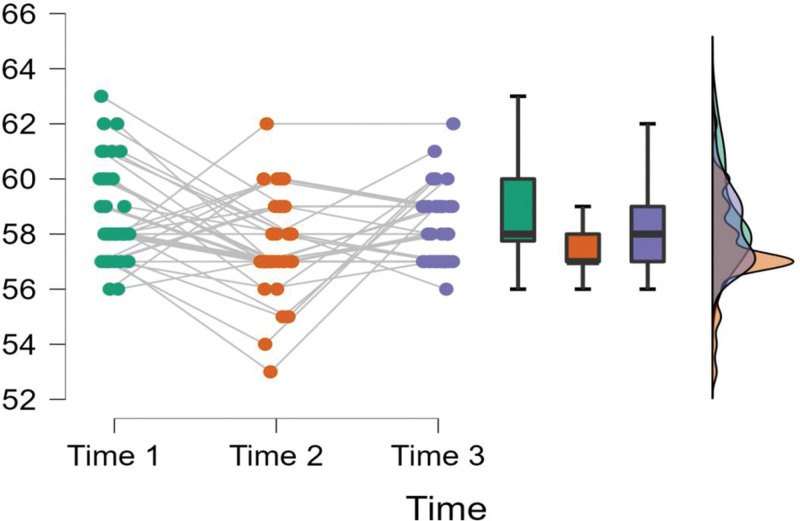
Raincloud plots burnout scores across time for control group.

Figure 3 further indicates that the individual burnout scores of the participants in the treatment group were clustered around the mean, strengthening the outcome regarding the effectiveness of the treatment. On the other hand, Figure 4 shows that the burnout outcomes of the control group did not vary significantly across time 1, 2, and 3. Also, the individual mean scores of the participants in the control group were more dispersed compared to the treatment group (See Fig. 4).

## 4. Discussion

This study aimed to identify whether an online intervention (online REBT) would be effective in reducing job burnout among Nigerian university history lecturers. It was found that the online REBT intervention was effective in reducing symptoms of job burnout in history lecturers. The results of the study show a significant effect of group, time, and time and group interaction on history lecturers’ burnout. There was a high level of job burnout among history lecturers, which is in accordance with reports by Fernández-Suárez et al^[33]^ This finding is consistent with those of the following researchers^[31,34–38]^ who have identified REBT as an effective intervention for burnout. According to the findings of this study, online intervention is effective at reducing job-burnout, confirming the findings of Jonas et al^[39]^ who found that online intervention helped participants reduce their symptoms of burnout. This study’s findings agree with those of Lanz et al^[40]^ who found a significant decrease in burnout levels of participants in the intervention group using an online intervention. Other related Nigerian studies showed that blended REBT which involved online and face-to-face interventions was efficacious in reducing stress.^[41,42]^ The present study strengthens the outcomes of the prior studies stated above. Further, the outcome of the present study also confirms the positive effects of using telegram as an online intervention tool; this is in line with findings of Tavakoli et al^[43]^ and Eseadi.^[44]^ The research implication of this study is that future investigators will be able to use this online psychological intervention model of REBT to alleviate the problem of job burnout experienced by university history lecturers. The authors also hope that this study can serve as a springboard for further research to determine whether or not online REBT intervention is a practical treatment approach for burnout among other university employees. As for policy implication, this study suggests that universities should adopt a workplace mental health policy that supports the use of online REBT to combat the symptoms of burnout that history lecturers encounter. In addition, the study emphasizes the importance of universities demonstrating a commitment to the lecturers’ mental health since this is crucial for human resource development and organizational growth. In light of the fact that recent studies have consistently shown the negative consequences of job burnout among employees including low job satisfaction, high turnover intentions, and poor teacher-student relationships,^[8–11]^ these policy actions are necessary.

A limitation of the present study is that it did not include any history lecturers from private universities; another limitation was the fluctuating Internet connections experienced sometimes during the online intervention, leading to some delays in the delivery of some content. This study also has geographical scope limitation as it was limited to only public university history lecturers in Southeast Nigeria. However, the study presents a modality for management of burnout condition in history lecturers’ population which is an understudied group. The study adds to the existing literature by adopting a timely approach to mental health intervention, given the socio-economic and occupational situation of the world and of Nigeria in particular at the time of the study. The study has presented a novel set of data that prioritize mental health of a select group of university lecturers.

## 5. Conclusion

In this study, we aimed to assess the effect of online rational emotive behavior therapy intervention on job burnout of Nigerian university history lecturers. This study concluded that the online REBT intervention reduces job burnout among history lecturers. The current study paves way for future studies to validate the efficacy of online rational emotive behavior therapy intervention among other employees who struggle with burnout. It presents a viable model for the treatment of occupational burnout that will be of research interest to occupational therapists, especially with the increasing surge into online therapeutic modalities. Practically, this study has revealed that online intervention reduces lecturers’ burnout; and theoretically, it revealed that the theory of REBT can be applied in this context. School mental health practitioners in universities can leverage online intervention for burnout treatment.

## Author contributions

**Conceptualization:** Chiedu Eseadi, Zadrian Ardi, Vera Victor-Aigbodion, Roland Ndille, Hero Usiomoefo Obasuyi, Shulamite Ebere Ogbuabor.

**Data curation:** Chiedu Eseadi, Zadrian Ardi, Vera Victor-Aigbodion, Roland Ndille, Hero Usiomoefo Obasuyi, Shulamite Ebere Ogbuabor.

**Formal analysis:** Chiedu Eseadi, Zadrian Ardi, Vera Victor-Aigbodion, Roland Ndille, Hero Usiomoefo Obasuyi, Shulamite Ebere Ogbuabor.

**Funding acquisition:** Chiedu Eseadi, Zadrian Ardi, Vera Victor-Aigbodion, Roland Ndille, Hero Usiomoefo Obasuyi, Shulamite Ebere Ogbuabor.

**Investigation:** Chiedu Eseadi, Zadrian Ardi, Vera Victor-Aigbodion, Roland Ndille, Hero Usiomoefo Obasuyi, Shulamite Ebere Ogbuabor.

**Methodology:** Chiedu Eseadi, Zadrian Ardi, Vera Victor-Aigbodion, Roland Ndille, Hero Usiomoefo Obasuyi, Shulamite Ebere Ogbuabor.

**Project administration:** Chiedu Eseadi, Zadrian Ardi, Vera Victor-Aigbodion, Roland Ndille, Hero Usiomoefo Obasuyi

**Software:** Chiedu Eseadi, Zadrian Ardi, Vera Victor-Aigbodion, Roland Ndille, Hero Usiomoefo Obasuyi, Shulamite Ebere Ogbuabor.

**Supervision:** Chiedu Eseadi, Zadrian Ardi, Vera Victor-Aigbodion, Roland Ndille, Hero Usiomoefo Obasuyi, Shulamite Ebere Ogbuabor.

**Validation:** Chiedu Eseadi, Zadrian Ardi, Vera Victor-Aigbodion, Roland Ndille, Hero Usiomoefo Obasuyi, Shulamite Ebere Ogbuabor.

**Writing – original draft:** Chiedu Eseadi, Zadrian Ardi, Vera Victor-Aigbodion, Roland Ndille, Hero Usiomoefo Obasuyi, Shulamite Ebere Ogbuabor.

**Writing – review & editing:** Chiedu Eseadi, Zadrian Ardi, Vera Victor-Aigbodion, Roland Ndille, Hero Usiomoefo Obasuyi, Shulamite Ebere Ogbuabor.
